# Supplementary material for: No evidence that hominin dispersal across Eurasia was part of a wider turnover in mammal distributions
Source: Nat Commun. 2026 Apr 17;17:3575. doi: 10.1038/s41467-026-71648-w (PMC13090383; doi:10.1038/s41467-026-71648-w)
Supplement: Supplementary file 1 — Supplementary information [file 41467_2026_71648_MOESM1_ESM.pdf]

Supplementary Information

No evidence that hominin dispersal across Eurasia was part of a wider turnover in mammal distributions

Jijia Sun[1,2*], Ignacio de la Torre[3], Faysal Bibi[1*]

[1]Museum für Naturkunde, Leibniz Institute for Evolution and Biodiversity Science, Invalidenstraße 42, 10115, Berlin, Germany.

[2]Faculty of Life Sciences, Humboldt-Universität zu Berlin, Unter den Linden 6, 10099 Berlin, Germany

[3]Pleistocene Archaeology Lab, Instituto de Historia, CSIC-National Research Council, Albasanz, 26-28, 28037, Madrid, Spain.

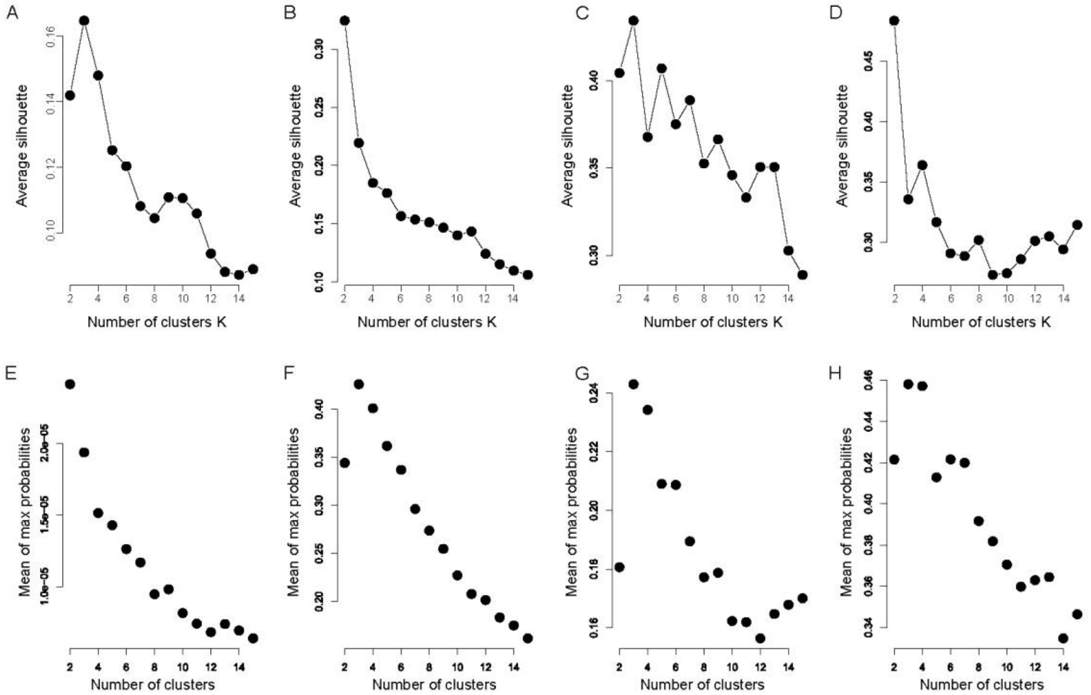

Supplementary Fig. 1 Results of silhouette coefficient (A-D) and AMD (E-H) analyses to determine the best number of clusters in the taxonomic and functional data. Silhouette coefficient analysis of **A:** fossil taxonomic communities (k=3). **B:** fossil functional communities (k=2). **C:** extant taxonomic communities (k=3). **D:** extant functional communities (k=2). AMD (Average Membership Degree) analyses of **E:** fossil taxonomic data (2 clusters). **F:** fossil functional data (3 clusters). **G:** extant taxonomic data (3 clusters). **H:** extant functional data (3 clusters). The silhouette coefficient and AMD analyses shown in panels A, C, F, G, and H identified 3 as the optimal number of clusters. Thus, 5 of the 8 analyses supported k=3 as the best choice. Based on this result, we used k=3 for all analyses throughout the study.

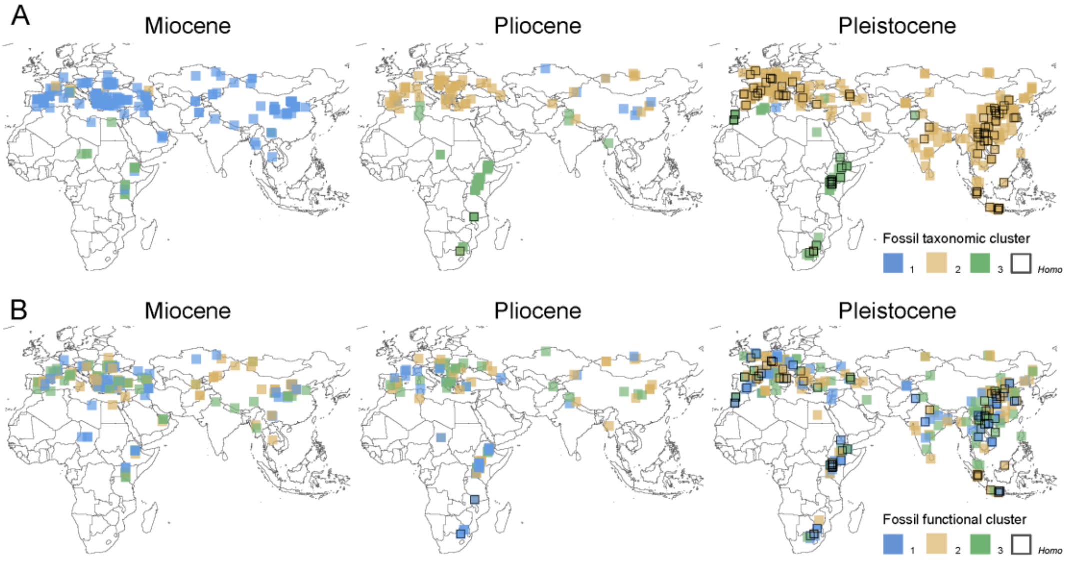

Supplementary Fig. 2 Fossil taxonomic and functional clusters visualized by epoch. Same data as Fig. 1 but plotted by epoch. A: fossil taxonomic clusters plotted by epoch.

B: fossil functional clusters. As in Fig. 1, there is a strong taxonomic separation of Eurasian and African faunas, and no geographic patterning of functional structure in fossil data. The map used in this study was generated using the rnaturalearth package in R.

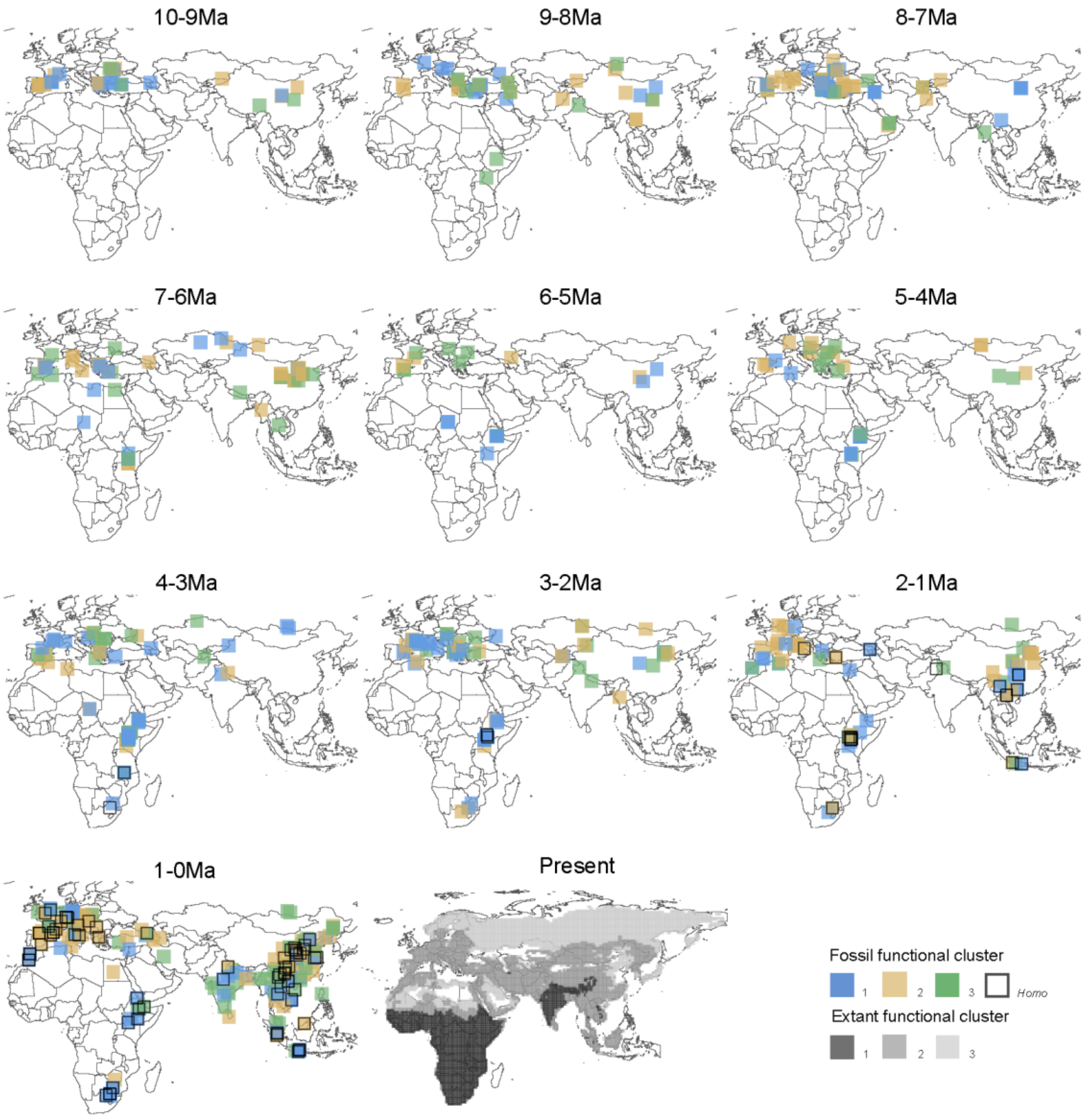

Supplementary Fig. 3 Fossil and extant functional clustering based on two traits (body mass, diet)

The results are similar to those of the analysis with all three traits (Fig. 1B). The map used in this study was generated using the rnaturalearth package in R.

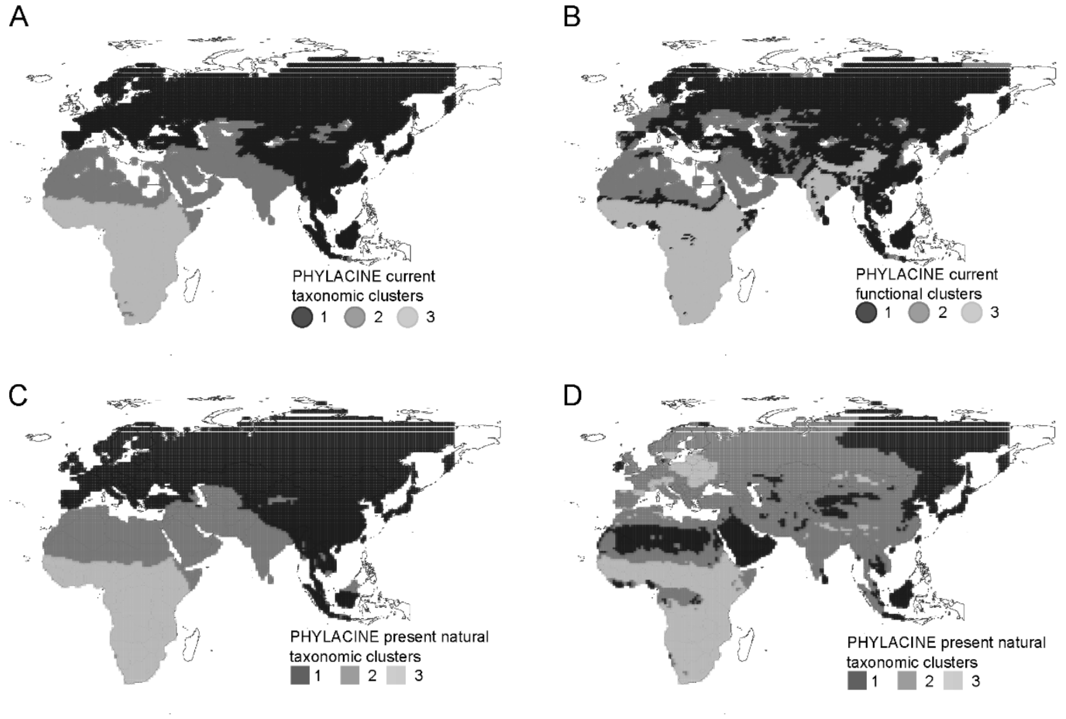

Supplementary Fig. 4 Extant taxonomic and functional clustering based on current and present natural ranges from PHYLACINE

A: extant taxonomic clustering based on current species ranges. **B:** extant functional clustering based on current species ranges. **C:** extant taxonomic clustering based on present natural ranges. Present natural ranges represent estimates of where species would live without anthropogenic pressures[1]. **D:** extant functional clustering based on present natural ranges. As in the IUCN dataset (Fig. 1), both the taxonomic and functional data show strong geographic structure. The map used in this study was generated using the rnaturalearth package in R.

Supplementary References

1. Faurby, S. *et al.* PHYLACINE 1.2: The Phylogenetic Atlas of Mammal Macroecology. *Ecology* **99**, 2626–2626 (2018).
