# Supplementary material for: No evidence that hominin dispersal across Eurasia was part of a wider turnover in mammal distributions
Source: Nat Commun. 2026 Apr 17;17:3575. doi: 10.1038/s41467-026-71648-w (PMC13090383; doi:10.1038/s41467-026-71648-w)
Supplement: Supplementary file 2 — Decription of Additional Supplementary Files [file 41467_2026_71648_MOESM2_ESM.pdf]

Supplementary Data 1. Raw fossil sites information in this study

The dataset includes site name, coordinates (longitude and latitude), continent, country, maximum age, minimum age and faunal composition in Eurasia and Africa within 10Ma.

Supplementary Data 2. Raw trait data in this study

The dataset includes the trait data (body mass, diet and locomotion) type and the sources for both fossil and extant genera in this study.

Supplementary Data 3. Synonyms in this study

The dataset includes synonyms used in this study.
